# How Do Portuguese Care Providers Address Disability and LGBT Identity in Their Work?

**DOI:** 10.3390/healthcare14081026

**Published:** 2026-04-13

**Authors:** Inês Soares, Ana R. Pinho, Liliana Rodrigues, Catarina Maria Rêgo-Moreira, Conceição Nogueira

**Affiliations:** Faculty of Psychology and Education Sciences, University of Porto, 4200-135 Porto, Portugal; psic.anapinho@gmail.com (A.R.P.); frodrigues.liliana@gmail.com (L.R.);

**Keywords:** LGBT, qualitative analysis, disability, professionals, care practices

## Abstract

Despite growing interest in the sexuality and gender identity of people with disabilities (PWD), this topic remains underexplored in both research and institutional policies, owing to prevailing views that ignore PWD sexual life. This contributes to the invisibility of individuals who identify as lesbian, gay, bisexual, and trans (LGBT) and to inadequate attention to the specific needs of LGBT people with disabilities (LGBT PWD). **Background/Objectives**: Given the lack of Portuguese studies that examine the intersection of LGBT and disability identities, this study aimed to understand professionals’ attitudes and practices toward PWD regarding sexuality and LGBT belonging. **Methods**: We conducted qualitative research using semi-structured interviews with eleven professionals (two psychologists, three occupational therapists, and six personal assistants). We analyzed the data using reflexive thematic analysis. **Results**: Key findings highlight professionals’ limited knowledge, prevailing cis-heteronormative attitudes, and emerging affirmative practices. **Conclusions**: Training and institutional changes are needed to make services more inclusive and responsive to the needs of LGBT PWD.

## 1. Introduction

People with disabilities (PWD) (the project originally used the term “functional diversity”; however, this manuscript uses “disability” to reach a broader audience while maintaining a person-first approach in line with APA guidance. Functional diversity (FD) is a term coined by the Spanish community Foro de Vida Independiente Y Divertad ([1]. It focuses on the discrimination and erasure of forms of functioning that deviate from the socially established norm and emphasizes the non-accommodation of these people and their way of functioning, rather than focusing on the individual while distancing itself from the negative connotation associated with the word “disability”. Functionally diverse people include “those who have long-term physical, mental, intellectual or sensory impairments which in interaction with various barriers may hinder their full and effective participation in society on an equal basis with others” [2]) (PWD) are frequently at risk of having their sexual behaviors misinterpreted as disability-related behavioral problems [3,4], contributing to their desexualisation. This often produces a dichotomous view that frames PWD as either *asexual* (Despite the use of asexual in various articles, this does not refer to the sexual orientation (internal process) but rather to the perception (and often desexualization) of these persons as sexless (external process), meaning they are seen as lacking sexual desire, activity, attractiveness or even gender in some cases [1]) or *hypersexual*, reflecting a broader failure to recognize their sexuality [5,6,7,8]. Even when their sexual and/or gender identity is acknowledged, it is usually shaped by cis-heteronormative (that poses cis-heterosexuality as “the fundamental and normative sexual orientation that dominates nearly all social and institutional spaces” [9]) assumptions [7,10,11,12]. Combined with homophobia [13,14], these attitudes mean that spaces aiming to be inclusive often fall short, particularly in accommodating lesbians, gays, bisexuals, and trans (LGBT) [8] individuals.

This marginalization of LGBT people with disabilities [7,15,16] contributes to documented health disparities [17] and to the absence of tailored policies and services [10,13]. It stems from the dominance of disability over other identities [12,16], the assumption that individuals are cis-heterosexual (Cis-heterosexuality consists of the assumption that there are only two genders—male and female—analogous to the sex assigned at birth, that they are opposites and only sexually attracted to each other) [8] and the reluctance of service users to disclose their sexual orientation or gender identity due to fears of jeopardizing support services [10]. Some studies report that Portuguese LGBT individuals refrain from disclosing their sexual orientation to health professionals [18], despite seeking information related to sexuality [19].

Families’ and support services’ stigma toward LGBT identities can shape PWD’s sexuality, gender expression, and related beliefs, possibly causing their desires and needs to be dismissed [7,8]. This influence is intensified by PWD’s limited access to broader third places [5,20,21]—that are neither home nor institutions. Misinformation can be used to control PWD’s behavior, undermine their privacy and self-expression [6], potentially hindering identity development [4,22], and fostering risky behaviors.

McGrath et al. [19] listed the main topics professionals address when discussing sexuality with PWD: the impact of diagnosis and medication on sexuality, responses to patient concerns, resumption of sexual activity after acute episodes, and guidance on adapting sexual practices to new health conditions. Only two studies in their review [19] reported broader dimensions of sexuality, namely self-concept and self-image.

The literature also documents professionals’ attitudes toward the sexuality of LGBT PWD. Commonly reported issues include: shame and discomfort among staff when discussing sexuality with clients [7,8,13,23]; viewing the topic as private, vulnerable [8,23] and potentially problematic [3]; lack of initiative in introducing the topic [7,8] and reliance on the user to raise it; fear of retaliation from the family of origin [8]; lack of training, knowledge and cultural literacy regarding sexuality and LGBT issues [7,13,19,23]; limited contact time [7,23]; absence of organizational guidelines about what should be addressed and how, despite professionals’ interest in such guidance [7,13]; belief that sexuality falls outside their abilities and responsibilities; and professionals’ own religious and cultural limitations or prohibitions [7]. Smith et al. [7] further note that some staff members believe that non-normative gender expressions could increase vulnerability to sexual violence. Dyer and das Nair [23] highlight that some of these attitudes are even more prevalent when discussing non-normative sexual orientations and gender identities regarding people with intellectual disabilities. Although research in Portugal on LGBT PWD is limited, de Sá [6] found that professionals consider sexual education insufficiently accessible.

It is argued that these attitudes contribute to the silencing of LGBT PWD’s sexuality, cis-heteronormative attitudes, and create barriers to inclusion and self-determination [14,24].

Health services and professionals providing support to PWD should operate with clinical and ethical competence [25], particularly given the elevated risk of mental health challenges among LGBT people [4,19,26,27,28]. These spaces have the potential to have a positive and regenerative impact on LGBT PWD’s lives [29] by fostering collaborative practices [20,30] and drawing on scientific evidence [14]. Moreover, staff members’ protective yet restrictive stance contradict service users’ expressed desire to discuss sexuality and relationships in safe, personalized ways [7,8].

Research identifies several measures to improve practice, such as facilitating coming out [31] and promoting the development of sexual and affective communication skills [6]; providing training to accommodate trans people [3,7,32]; understanding the specific challenges LGBT people face (sexual minority stress, alienation, adjustment disorder issues, and how specific therapies can interfere with sex reassignment treatments) [9,30]; and educating staff about sexual identity, relationships and accessible practices. Additional recommendations include recognizing negative attitudes and cis-heteronormative patterns; discussing vulnerability, capacity, consent, abuse and exploitation; sharing knowledge about safe practices and intimacy; using neutral language; including partners in rehabilitation; promoting privacy; using inclusive institutional materials; listing available activist groups [33]; and defining ways to report discriminatory practices [10].

Despite growing scholarly and social attention to sexual and gender minorities and disabilities [34], research addressing their intersection remains limited, particularly in Portugal. Attending to the specificities of this population is essential, as individuals experience distinct and overlapping forms of oppression [12,34,35,36]. It is, therefore, important to examine how professionals perceive the sexual orientation and gender identity of LGBT PWD and to identify strategies that reduce discrimination and foster well-being [37].

Although research indicates growing acceptance of non-normative sexual orientations in Portugal, persistent prejudices toward non-normative gender identities remain. While disability-related vulnerability is acknowledged, structural responses particularly within health services are still limited [38]. Moreover, empirical studies that examine the intersection of these categories are largely absent.

Given Portugal’s commitment to protecting the rights of minority groups (the mention of minority groups does not refer to the size of this population but to the power they hold within the general population. These are considered marginalized identities, classified as outside of the social norm [9]) [39] through national plans, conventions, and strategies, this study aims to explore how sexual and civil rights are protected by the professionals who provide services to LGBT PWD.

The present study aims to: (Aim 1) explore existing practices in working with LGBT PW; (Aim 2) understand the needs and difficulties associated with LGBT and disability issues in the context where professionals work; and (Aim 3) identify suggestions for improvement.

### Research Questions

Following the study objectives, we formulated the following research questions: (Q1) In what ways do professionals know about and are sensitized to LGBT issues when working with PWD? (Q2) How do professionals perceive the non-normative sexual orientation and/or gender identity of PWD? (Q3) What are professionals’ discourses and practices regarding non-normative sexual orientation and gender identity? (Q4) What are the difficulties or needs for change in providing services to LGBT PWD identified by professionals? (Q5) What are the participants’ suggestions for improvement in the work with LGBT PWD?

## 2. Materials and Methods

### 2.1. Participants

We defined the inclusion criteria as the current provision of professional care services to people with disabilities within the partner associations involved in the project (which introduced the study’s aims and ensured a shared baseline of knowledge about disability and LGBT+ issues). Eligible participants were required to be actively working with PWD and to have attended the project’s preparatory sessions. Exclusion criteria included not working with PWD at the time of recruitment and not belonging to the participating associations. Recruitment followed a convenience strategy [40,41] through the social networks of the associations responsible for the project (see Appendix A).

We interviewed eleven professionals. One person identified as trans non-binary, one as male, and nine as female. Ages ranged from 27 to 55. Two participants were psychologists (PSY), three were occupational therapists (OT) and six were personal care assistants (PCA). Six participants had contact with LGBT people in their work environment, and five did not. Those without prior experience with LGBT people were included due to the limited availability of LGBT PWD within the participating associations.

Sociodemographic data were collected at the end of the interviews and can be consulted in Table 1.

### 2.2. Data Collection

Data were collected through semi-structured interviews (see Appendix B) [40,41]. Data collection ceased because the allotted time period had elapsed. Interviews were conducted online via Zoom, in 2022, and lasted an average of 35 min. All interviews were audio-recorded and later transcribed by the interviewer to ensure anonymity.

### 2.3. Data Analysis

We used Reflexive Thematic Analysis (RTA) [40,41] to analyze the data, following its six steps (familiarizing ourselves with the data, generating initial codes, which are collated into candidate themes, then reviewing and refining themes, naming each theme, and producing the report). RTA offers flexibility to examine implicit meanings, identify patterns across interviewees, and map convergent and divergent perspectives in relation to the literature. The analysis was informed by a social constructionist perspective to understand how experiences and meanings are shaped by, and expressed through, socially reproduced discourse(s) [40,41]. We adopted an inductive-deductive approach, as themes were informed both by the existing literature and by new insights emerging from the interviews.

## 3. Analysis and Discussion

In this section, we present the four themes that emerged from the data—(i) attitudes and practices of professionals; (ii) specific challenges working with LGBT PWD; (iii) cis-heteronormativity; and (iv) need for training—together with their subthemes, codes, illustrative excerpts, and narrative interpretation. These themes interconnect through a shared pattern, the central organizer, *Practices of sexual and gender expression containment.*

**Figure 1:**
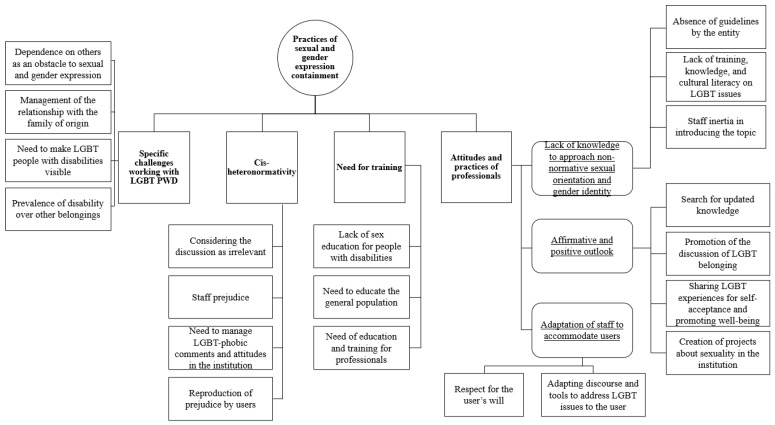
Thematic map analysis.

These practices are based on our interpretation, resulting from legislation, institutional policies (and lack thereof), and personal beliefs about what is acceptable or not. Figure 1 displays the thematic analysis map, which outlines the four themes (in bold formatting), their subthemes (underlined) and codes.

### 3.1. Theme I. Attitudes and Practices of Professionals

This theme explores present and future practices professionals adopt in their work. Even though nearly half of the interviewees have no prior experience with LGBT PWD (to the best of their knowledge), they still contemplate how they would engage with this population. We drew out three sub-themes: (i) *lack of knowledge to approach the theme*, which consigns the professional to passivity, subtheme (ii) *affirmative and positive outlook* and (iii) *adaptation of staff to accommodate users,* which denote a more active role on the part of the staff.

#### 3.1.1. Sub-Theme I. Lack of Knowledge to Approach Non-Normative Sexual Orientation and Gender Identity

This sub-theme highlights that professionals lack knowledge about the LGBT community due to insufficient cultural literacy, scientific knowledge, academic training, and workplace guidance. These gaps make it difficult for them to address the topic, contribute to the marginalization of LGBT PWD, and limit the support they provide.

Sub-theme (i) reflects C1. *Absence of guidelines by the entity*, which results in inadequate support for both users and professionals. Without clear procedures, sexuality is addressed only when unavoidable and mainly in conflict-resolution contexts.

*“We never talk about that [sexuality and LGBT belonging] in the courses, so we go a lot into the conflict part, the support part, how we have to deal with a lot of situations”* (E, PCA)

Although professionals express interest in having structured procedures and inclusive materials [8,13], these resources remain unavailable, creating uncertainty about what topics to address and how to approach them [7,10,28]. Clear guidelines could improve interactions with families [23] and help address unmet needs [20].

The lack of internal policies for addressing LGBT issues with PWD reinforces a C2. *Lack of training, knowledge, and cultural literacy on LGBT issues*, leading professionals to rely on informal sources and media for information.

*“Very little [knowledge], quite honestly, almost none (…) We have some contact on television, but I have never had any education or training”* (E, PCA)

This absence of policies [7,8,13,19], combined with the belief that only specialists should address these topics, may result in professionals seeking advice from colleagues or referring users elsewhere [25]. Time constraints, heavy workload, and the absence of guidelines by the entity further limit training opportunities. These conditions contribute to C3. *Staff inertia in introducing the topic*, marked by reluctance to introduce the topic and low confidence, often accompanied by shame and discomfort [7,13,19,28]. Research shows that only a minority of professionals address sexuality, despite recognizing its significance for health and well-being [19,23].

*“Perhaps not so many opportunities are given to people with disabilities, and with that… there you have it, because we still lack information and are very closed off, there is still a lot of shame in talking, in discussing these issues”* (B, PCA)

#### 3.1.2. Sub-Theme II. Affirmative and Positive Outlook

This sub-theme shows that some professionals actively sought information, welcomed the topic when raised by users, clarified doubts, and encouraged discussions. Institutions also took steps to address sexuality and LGBT identity in broader group settings involving staff, users, and, at times, families.

Professionals engaged in C4. *Search for updated knowledge*, a core element of competent practice. Several participants independently sought information on LGBT health and clinical issues through scientific, recreational, and cultural sources [10,25,42].

*“I had already read some things on the subject and seen films and read some articles, even regarding transsexualities, operations, hormonal aspects, right? About gender identification”* (H, PCA)

Professionals with extensive training in sexuality showed greater interest and knowledge about diverse sexual orientations and gender identities, which improved their attitudes and comfort level, consistent with the existing literature [6,19,34,35]. Those who invested most in learning were also the most engaged in the C5. *Promotion of the discussion of LGBT belonging*, recognizing its positive impact and the importance of supporting identity development [4]. Similar studies also found that only professionals already interested in LGBT issues tend to initiate these conversations [8].

*“At the time, I got to talk to the person who was responsible for that area so that they could explore in a group setting with the other peers, and everything, that matter [non-normative gender identity of a user]. And also help him to, maybe, define what was happening or to understand himself a little bit better”* (I, PSY)

These discussions should avoid judgment and bias [34] and address the contextual barriers and stressors experienced by LGBT PWD due to social oppression. Such conversations can shape attitudes, strengthen critical thinking, and reduce feelings of inadequacy [26] and risky behaviors [10]. Staff should address vulnerability, consent, abuse, and exploitation [10], fostering resilience and creating safe, inclusive environments [9].

Professionals aimed for C6. *Sharing LGBT experiences for self-acceptance and promoting well-being*, reporting positive outcomes from these conversations.

*“But still it is very good that he did [coming out] and had that openness from his colleagues, and really, the reaction of the group wasn’t bad, it wasn’t… it was positive. And I think it may have been good for him, too, to experience acceptance (…) also to start to have a little bit of acceptance within himself”* (I, PSY)

Because coming out requires skills that people with intellectual disabilities may not have practiced, staff should provide adequate support [7]. Promoting acceptance, diversity [34], empowerment, and well-being [4] strengthens therapeutic alliance and self-esteem [35].

Finally, some professionals initiated C7. *Creation of projects about sexuality in the institution*. Although not exclusively focused on LGBT concerns, diversity-oriented projects positively influence self-concept, identity, belonging, pride, resilience, and access to practical information. Such initiatives can compensate for the *Absence of guidelines by the entity* and demonstrate innovative approaches to inclusion [29,34,35]. One participant noted that inclusive services improve access to information and education and involve PWD in the design of group activities and rules [29].

*“I started with the sexuality project… and began to explore this with them (…) I did interventions with caregivers and even professionals”* (I, PSY)

#### 3.1.3. Sub-Theme III. Adaptation of Staff to Accommodate Users

This sub-theme delves into the staff’s ability to adapt communication and accommodate users, including respecting users’ preferences and values even when staff hold negative beliefs about LGBT identities. The emphasis falls on prioritizing the individual over personal biases.

The C8. *Respect for the user’s will* aligns with the United Nations (UN) [2], which affirms the importance of autonomy, independence, and respect for personal choices [10], including clothing, gender expression [28], and the creation of safe spaces [35].

*“Of course, there are certain situations that cause us strangeness because we are not used to contacting, to deal with, but I would do my best and help the person in whatever they needed”* (B, PCA)

Professionals who take an active role in discussing LGBT belonging with PWD emphasize the need for C9. *Adapting discourse and tools to address LGBT issues to the user* so that information is accessible and tailored to each person’s disability. The literature supports adaptive approaches to physical intimacy and sexual expression [10] and highlights the importance of equipping staff with communication skills [19] to ensure a clear exchange and comprehension.

*“We have two cases here… it is relevant [to approach LGBT topics in the context where you work] (…), it depends on the case, and of course, it could be something beneficial, it depends if we were talking to young people with a slight disability, right? Now we’re not going to be talking to a group of users with a moderate or severe disability who are not going to understand what I’m talking about (…). We should have a simpler language, shouldn’t we?”* (G, OT)

### 3.2. Theme II. Specific Challenges Working with LGBT PWD

This theme explores barriers hindering users’ full sexual and gender expression, including dependence on third parties and limited social networks. The dominance of disability over other identity categories underscores the need to increase the visibility of LGBT PWD, who often lack representation.

Restricted social circles, limited access to information, and confinement to private or institutional spaces create a C10. *Dependence on others as an obstacle to sexual and gender expression*. Participants noted that these constraints, combined with family influence, shape users’ values, and behaviors. As a result, caregiver and family stigma often determines the level of support provided, rather than the individual’s actual needs [6,7,8,12].

*“Yes, [PWD] have even more barriers, because… especially if they are very dependent on other people, it becomes even more complicated (…) if they are dependent on someone else to make them… if the person does not respect their ideas, well, it is much more complicated”* (B, PCA)

Although the UN [2] advocates that autonomy and independence should be ensured to the greatest extent possible, paternalistic restrictions persist [7,8].

Given that families may perpetuate abuse and discrimination related to both disability and LGBT belonging [20], C11. *Management of the relationship with the family of origin* becomes essential to safeguarding the freedom and individuality of LGBT PWD, particularly in conservative cultural contexts such as Portugal. This challenge intensifies when working with minors or when boundaries with families are unclear.

*“It is quite important, and it is not very… sometimes it is not very easy [to address the topic] (…) it is important to keep an optimal distance, we can’t get too close, nor can we be too distant, well, my goal is to be a support, an aid. But we also can’t impose ourselves on the family, on the person, right? And to encourage or to withdraw… it has to come from them”* (H, PCA)

Professionals and users often anticipate negative family reactions to discussions about LGBT topics and fear potential consequences [8,9,13,35]. Nonetheless, professionals can assess risks and determine appropriate ways to approach these topics [8], ensuring user safety while promoting dialog and acceptance.

Interviewees also stressed the C12. *Need to make LGBT people with disabilities visible*, as they often face discrimination both within the disability and the LGBT communities [14,29]. Professionals highlighted the importance of amplifying LGBT PWD’s voices, supporting their participation in community activities [33], and involving them in policy and program development [2]. Contact with other LGBT individuals can reduce loneliness and nurture positive identity formation [7,10,35].

*“It makes me very sad that we still do not give the word to those to whom it should be given to”* (I, PSY)

These issues converge in C13. *Prevalence of disability over other belongings*, which affects autonomy and freedom of expression. This prevalence appeared in professionals’ speeches, which often focused solely on disability even when questions explicitly addressed sexuality or LGBT identity. Such responses may reflect unfamiliarity with LGBT belonging.

*“Portuguese society is not a very open society (…) it is still a bit conservative (…) it is not accepted in many families, isn’t it? Homosexuality, right, changing gender, whatever… it is a deviation, isn’t it? Maybe they see it as a deviation, (…) we do not want to create problems, even more, for that boy or girl”* (H, PCA)

The C13. *Prevalence of disability over other belongings* aligns with research showing that stigma and shame surrounding disability overshadow gender identity and sexual orientation [10,12], downplaying non-normative aspects for easier acceptance [8]. This devaluation can result in the exclusion of LGBT PWD from the LGBT community [35], reinforcing the need to recognize diversity within the disabled population as a priority [2].

### 3.3. Theme III. Cis-Heteronomativity

This theme highlights beliefs, practices, and challenges shaped by cis-heteronormativity, underscoring the need for change. The aforementioned passive stance reflects C14. *Considering the discussion as irrelevant*, a position rooted in cultural constraints that hinder open and inclusive dialog [7,10,12]. Such passivity can heighten users’ fear of coming out to professionals [35].

*“I never approached it, I never felt the need (…) As they were always well accepted by their peers, by the team, by everyone… it was never necessary to approach this subject with them”* (G, OT)

Reluctance to address LGBT issues can reinforce cis-heteronormativity [7,10,13], contradicting their expressed needs and identities, leading to unaddressed difficulties [29,35]. This can increase the risk of unsafe behaviors, due to limited knowledge about consent, assertiveness, and healthy practices [4].

Participants’ acknowledged and criticized C15. *Staff prejudice*, often stemming from the belief that LGBT identity is irrelevant to supporting PWD. Even unintentionally, this stance perpetuates discriminatory behaviors and rejects non-normative GI/SO.

*“I do not accept it for myself, but other people have their life, they have their… they think as they wish”* (K, PCA)

Despite users’ self-acceptance, LGBT identities continue to face external challenges [20,30], and organizations often overlook the barriers that limit LGBT PWD’s access to the broader LGBT community [29]. Addressing these biases through education is crucial, as prejudice undermines clinical competence and services quality [10,25].

Given the presence of prejudice, institutions must address the C16. *Need to manage LGBT-phobic comments and attitudes in the institution*. Some interviewees strive to promote user comfort, challenge colleagues’ attitudes and foster a culture of respect [10].

*“[work through LGBT phobic beliefs and behaviors with professionals] just so that, first, the behaviors wouldn’t be reproduced and, second, that [users] wouldn’t feel oppressed”* (I, PSY)

Research documents instances of staff-perpetrated abuse and discrimination against LGBT people [20,35], which compromises safety and heightens vulnerability [30]. Teams must confront their own prejudices and discriminatory behaviors [26]. Professionals also noted some C17. *Reproduction of prejudice by users*, often attributed to their social environments and upbringing [4]. This pattern highlights the need for institution-led efforts to counteract prejudice, potentially through inclusive sex education [31].

*“Among them, they do not discriminate against each other, neither for gender identity nor for sexual orientation (…) this is the majority. Of course, there is always one or two that we can see have a marked parental education, even by the type of comments they make, we realize that these are not their words, right? Even by the level of grammar construction and the type of vocabulary used, we realize that they are not… they heard this at home”* (J, OT)

### 3.4. Theme IV. Need for Training

The final theme reflects a shared recognition among staff of the need for training to improve service provision, strengthen the inclusion of LGBT PWD, and enhance their quality of life. Training should involve broad and diverse educational initiatives, including inclusive sex education for PWD, which can help prevent the reproduction of prejudiced beliefs among both staff and users. Within this theme, C18. *Lack of sex education for people with disabilities*, emerges as a central concern, highlighting the need for accessible programs that explicitly address LGBT topics.

*“I researched more interactive contents to really try to make it work and pull them in, because despite everything it is still a difficult subject… it is still inaccessible, they’re very annoying, these are things they’ve never heard of, never… (…) and, therefore, we really need to go to a really basic level and start from the bottom and do everything calmly. So I really looked for materials, books, games, to make things a little bit more interesting”* (I, PSY)

Although Portuguese professionals value sex education, a persistent gap remains when addressing the sexuality of PWD [6]. Closing this gap through evidence-based and inclusive information is crucial for strengthening self-esteem, fostering positive perceptions of relationships, developing decision-making skills [7], and ensuring rights and freedoms [2]. Interviewees also stressed the C19. *Need to educate the general population*, noting that broader education can help families and close social circles provide adequate support [3,7,30] and prevent the burden from falling solely on LGBT PWD. Deconstructing entrenched beliefs is necessary to promote harmony across social environments.

*“You have to reach all the places because they leave from there [institution] and then go to other systems, and other systems that reproduce behaviors that are not… not the most positive ones”* (I, PSY)

A key requirement for meeting users’ needs and creating welcoming environments is the C20. *Need of education and training for professionals*, especially given the previously identified lack of training and organizational guidance.

*“I think it is important to have some training, both for the users and for the personal assistants”* (D, PCA)

*“The problem is that there is no continuous work (…). We had your education session some time ago, now it will be in five years, I do not know, with luck”* (F, PSY)

Insufficient preparation often shifts the responsibility onto users to initiate conversations about LGBT issues [7,8,13,19]. In contrast, professionals who take an active role tend to promote well-being, acceptance, awareness, and quality of life for LGBT PWD, aligning with inclusive and affirmative practices [5,24,29,35]. This proactive stance is more common among professionals with greater experience, interest, and knowledge in LGBT and sexuality-related topics [6], although they represent a minority.

Professional training increases comfort in discussing LGBT issues and supports identity exploration [4,7,8]. It also helps professionals understand the specific challenges faced by LGBT individuals, enabling them to assess sexuality-related concerns within the context of sexual orientation and gender identity [10], while countering monolithic representations [9]. Training should be grounded in ethics and intersectionality [42] and promote clinical competence and LGBT-affirming care [25,26]. Relevant topics include accommodation for trans people [7,32], the psychological process of coming out, awareness of personal biases [24], the role of language in identity development, available resources, and strategies for addressing LGBT-phobic behaviors [22]. Comprehensive training for all staff ensures consistent knowledge and practices and requires sustained public investment to guarantee regular and updated sessions [14].

## 4. Conclusions

The findings show that professionals’ discourses and practices are still shaped by cis-heteronormativity and by a tendency to try and contain the sexual and gender expression of LGBT PWD, due to a lack of better knowledge. This reflects a broader pattern in which the sexual and bodily autonomy of PWD is not fully recognized, and where the absence of clear national guidelines lead institutions to prioritize prevention and conflict management over affirmative support [7,13]. Even so, several professionals demonstrated a genuine commitment to meeting users’ needs and, in some cases, introduced inclusive practices independently, suggesting that incremental change is emerging within services.

Although all interviewees expressed a desire to respect users’ self-expression, this may reflect an optimistic bias, particularly because many had no direct experience working with LGBT PWD. Limited contact may itself indicate barriers in service accessibility and inclusiveness [29]. As noted in previous research [8], “equal treatment” often translates into treating all users as if they were cis-heterosexual, thereby reproducing invisibility.

Across themes, professionals identified several structural and relational challenges: navigating family influence, providing inclusive sex education [31], increasing the visibility of LGBT PWD, and confronting dominant beliefs that prioritize disability over other identities. A hierarchy of recognition persists, with disability frequently overshadowing sexual orientation and gender identity, and with non-normative gender identities receiving the least attention [3,7,8].

Professionals’ training on sexuality and LGBT issues is crucial, as there is currently a lack of education, guidelines, and procedures in institutions. There is also a need to manage discriminatory behaviors [37] and provide safe environments [43].

Training emerged as a central need. Professionals lack structured education, institutional guidelines, and procedures for addressing sexuality and LGBT issues, and they must also manage discriminatory behaviors and ensure safe environments [37,43]. Given the documented experiences of microaggressions, stigma, and oppression among LGBT PWD [9], services must adopt affirmative, rights-based approaches [33] that recognize sexual and relational needs alongside housing, employment, and general health [13]. Such approaches can strengthen self-acceptance, reduce loneliness, and mitigate risks of depression, anxiety, and suicidal ideation [28].

This study has limitations. Translation may have reduced nuance, and preparatory sessions may have influenced responses toward social desirability. The sample did not include PWD, was predominantly female, and many participants had no experience with LGBT PWD. Participation may also have been biased toward those more open to LGBT issues. The study’s broad scope—across diverse disability types and LGBT identities—limits the specificity of conclusions. Another limitation is the study’s broadness—given the broad diversity within both the categories of disability (physical, intellectual, or sensory, also, if it is acquired or congenital) and the LGBT community (particularly regarding non-normative sexual orientations and gender identities) [3]. Nonetheless, the findings align with the existing literature and indicate that some professionals are already taking individual steps toward more inclusive practices.

Future research should center the voices of LGBT PWD, explore family perspectives, and evaluate the effectiveness of affirmative practices, particularly among younger populations. It is also essential to examine how different disability types intersect with diverse LGBT identities to better inform policy and practice. Future research should also examine how different professional roles shape attitudes and practices, as job-specific responsibilities may influence how sexuality and gender identity are addressed.

## Figures and Tables

**Table 1:** Participants’ sociodemographic characteristics.

Baseline Characteristic	*n*	%
Gender		
Female	9	81.8
Male	1	9.1
Trans non-binary	1	9.1
Nationality		
Portuguese	9	81.8
Brazilian	2	18.2
Highest Educational Level		
Middle school	1	9.1
High school	2	18.2
Bachelor’s degree	4	36.4
Master’s degree	4	36.4
Occupation		
Psychologist (PSY)	2	18.2
Occupational therapist (OT)	3	27.3
Personal Care Assistant * (PCA)	6	54.5
Contact with LGBT PWD in work practice		
Contact	6	54.5
No Contact	5	45.5

Note. N = 11. Participants were on average 39.4 years old (SD = 9.5). Ages ranged from 27 to 55 years old. Participants had on average 9.3 years of work experience (SD = 9.2). Years of experience ranged from 1 to 25 years. * One person had work experience as a teacher for special education in music, but at the time was practicing as a personal assistant.

**Figure A1:**
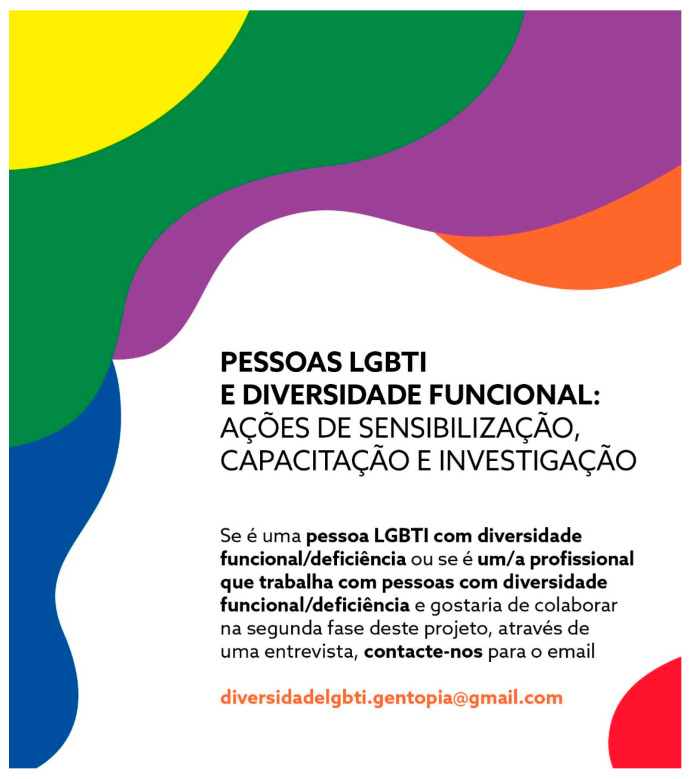
Leaflet shared to recruit participants.

## Data Availability

The data presented in this study are available upon request from the corresponding author due to privacy reasons.

## Appendix A. Leaflet

Translation: If you are a LGBTI person with functional diversity/disability or if you are a professional working with people with functional diversity/disability and you would like to collaborate in the second phase of this project, through an interview, please contact us at.

## Appendix B. Interview Script

General Aims

1. Explore existing practices in working with LGBT people with disabilities;

2. Understand the needs and difficulties associated with LGBT and disability issues in the context in which professionals work;

3. Identify suggestions for improvement.

Questions

1. Brief introduction/icebreaker: Who are you? What is your position and duties in the association?

2. Before attending the awareness-raising action promoted by this project, what did you know about this topic?

3. How relevant do you think it is to approach this theme in the context in which you work?

4. Have you ever had or dealt with a client/person who had any situation related to sexual orientation, gender identity, gender expression, and/or non-normative sexual characteristics? If yes, what were the situation/s? How was it addressed by the team? Tell me about it.

5. Did you feel/do you feel any difficulties? If yes, what difficulties did you feel (or do you feel) when dealing with this issue (when dealing with clients, with the family, with the team)?

5.1. What do you think would help overcome these difficulties?

6. Do you identify any needs in the association… regarding this topic? If yes, which ones? What could be improved?

7. And on a more general, public policy level, what do you think could contribute to an effective inclusion of LGBTI people with functional diversity/ disability?

8. We are reaching the end. Would you like to add anything we have not talked about or take something back from what you said?

9. How did you feel throughout this conversation?

10. Thanks
